# Synthesis of isobutanol using acetate as sole carbon source in *Escherichia coli*

**DOI:** 10.1186/s12934-023-02197-w

**Published:** 2023-09-27

**Authors:** Pengfei Gu, Shuo Zhao, Hao Niu, Chengwei Li, Shuixing Jiang, Hao Zhou, Qiang Li

**Affiliations:** 1 School of Biological Science and Technology, University of Jinan, Jinan, 250022 People’s Republic of China; 2 RZBC GROUP CO., LTD, Rizhao, 276800 Shandong China

**Keywords:** Isobutanol, *Escherichia coli*, Acetate, Metabolic engineering, Pyruvate

## Abstract

**Background:**

With concerns about depletion of fossil fuel and environmental pollution, synthesis of biofuels such as isobutanol from low-cost substrate by microbial cell factories has attracted more and more attention. As one of the most promising carbon sources instead of food resources, acetate can be utilized by versatile microbes and converted into numerous valuable chemicals.

**Results:**

An isobutanol synthetic pathway using acetate as sole carbon source was constructed in *E. coli*. Pyruvate was designed to be generated via acetyl-CoA by pyruvate-ferredoxin oxidoreductase YdbK or anaplerotic pathway. Overexpression of transhydrogenase and NAD kinase increased the isobutanol titer of recombinant *E. coli* from 121.21 mg/L to 131.5 mg/L under batch cultivation. Further optimization of acetate supplement concentration achieved 157.05 mg/L isobutanol accumulation in WY002, representing the highest isobutanol titer by using acetate as sole carbon source.

**Conclusions:**

The utilization of acetate as carbon source for microbial production of valuable chemicals such as isobutanol could reduce the consumption of food-based substrates and save production cost. Engineering strategies applied in this study will provide a useful reference for microbial production of pyruvate derived chemical compounds from acetate.

**Supplementary Information:**

The online version contains supplementary material available at 10.1186/s12934-023-02197-w.

## Introduction

In the area of microbial production of valuable compounds, selection of abundant and low-cost carbon sources is essential for products application and promotion [1]. Acetate, the second simplest carboxylic acid, is present in larger amounts in nature [2]. In general, acetate is a common by-product in microbial metabolism of sugars. In addition, acetate can be generated in anaerobic microbial fermentation of waste organic materials, such as lignocellulose depolymerization [3] and waste water treatment [4]. With properties of abundance, low price ($340 per metric ton) and easy utilization by versatile microbes, acetate become a feasible carbon source for microbial fermentation [5]. In fact, microbial production of value-added products from pure acetate or acetate-containing biorefinery stream has attract more and more attention over the past decade [6–8].

Many microorganisms can utilize acetate as alternative carbon source for cell growth, such as *Escherichia coli* [9], *Cryptococcus curvatus* [10], *Clostridium* sp. [11], and so on. Due to convenient genetic engineering tools and fast growth in cheap media, *E. coli* is a widely used host microorganisms in microbial fermentation industry [12]. In *E. coli*, acetate can be transformed into acetyl-CoA by AMP-forming acetyl CoA synthetase (Acs) or phosphotransacetylase/acetate kinase (Pta-AckA) pathways. The acetyl-CoA was then entered into the TCA cycle and glyoxylate cycle to supply energy and precursors for cell growth [13]. However, because of its toxicity and poor transformation efficiency compared with other carbon source, acetate is usually not a preferred carbon source. As a result, only a few products have been successfully synthesized in *E. coli* by using acetate as main carbon source, such as PHA [14, 15], ethanol [16], succinate [5, 17], fatty acid [18], itaconic acid [19], mevalonate [20], sweet protein [21], and glycolate [22].

Isobutanol (2-methyl-1-propanol) is a four-carbon alcohol which is a promising biofuel than ethanol due to its low hygroscopicity and vapor pressure, compatibility with existing engines, and high-energy capacity [23]. Therefore, isobutanol is considered as one of potential alternatives for traditional gasoline. In addition, isobutanol can also be served as a precursor for the production of a variety of polymers, paints, plastics and synthetic rubber [24]. In 2008, Atsumi et al. firstly reported isobutanol production in *E. coli* by employing native branched-chain amino acid biosynthesis pathway [25]. Subsequently, numerous recombinant *E. coli* with high yields and titers of isobutanol were obtained [26–28]. Apart from glucose, cellobiose [29], cellobionic acid [30] and sucrose [31] were also employed as substrate for synthesizing isobutanol in *E. coli*.

In this study, isobutanol production in *E. coli* with acetate as sole carbon source was investigated. Pyruvate was designed to be generated from acetate via acetyl-CoA by pyruvate-ferredoxin oxidoreductase YdbK or anaplerotic pathway. Overexpression of transhydrogenase and NAD kinase as well as optimization of acetate supplement concentration further increased the isobutanol titer of recombinant *E. coli* WY002 to 157.05 mg/L, representing the highest isobutanol titer by using acetate as sole carbon source.

## Results and discussion

### Construction of an isobutanol biosynthetic pathway from acetate in ***E. coli***

**Fig. 1:**
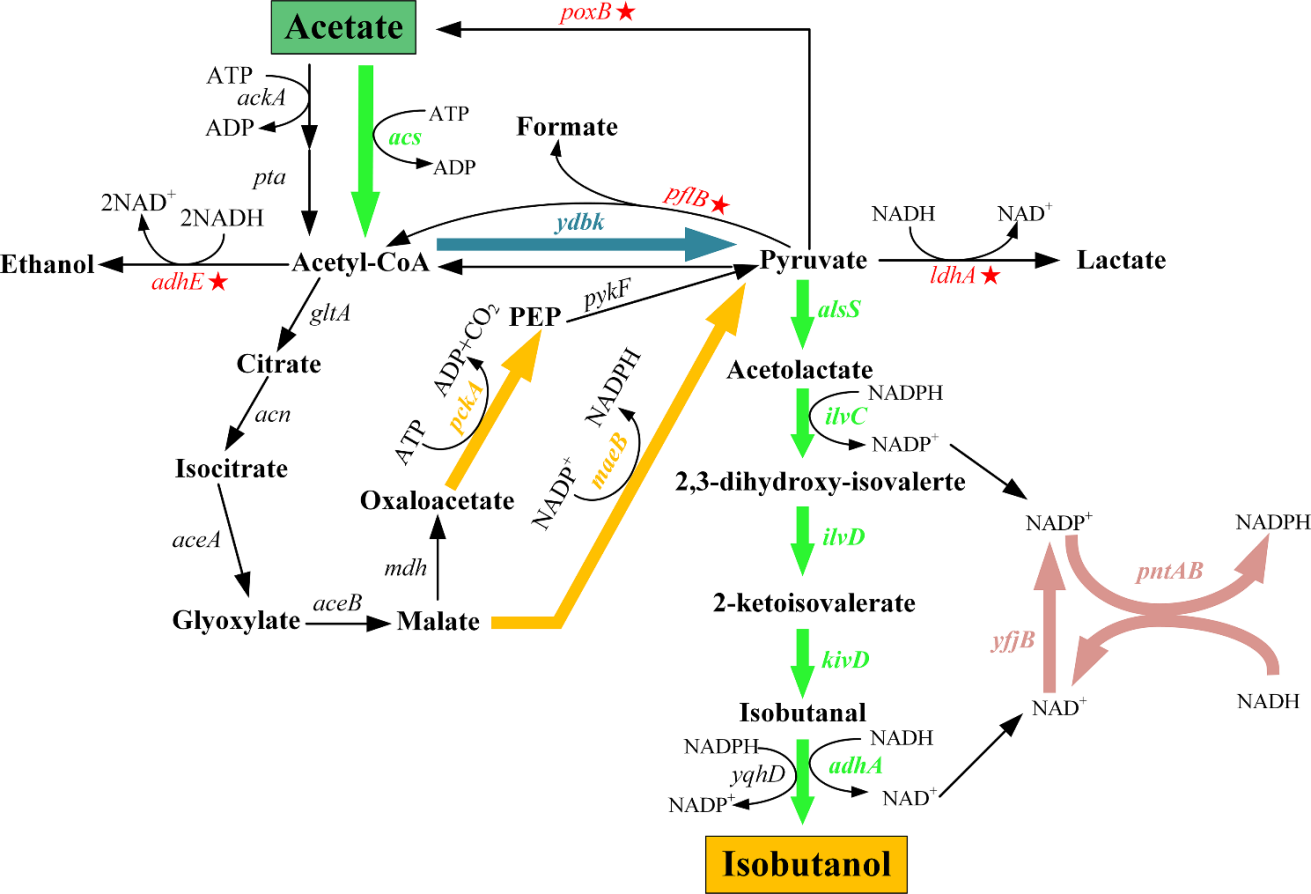
Constructed synthetic pathway of isobutanol from acetate in *E. coli*. The red stars indicate the genes that were deleted. The thick colored arrows indicate the increased flux by directly overexpressing the corresponding genes in plasmids

In previous studies, an effective isobutanol biosynthetic pathway had been created by combining branched-chain amino acids synthetic pathway from glucose and Ehrlich pathway with 2-keto-isovalerate serving as a precursor [25]. The *alaS* gene encoding acetolactate synthase from *Bacillus subtilis* was responsible for converting pyruvate to acetolactate. And then, 2,3-dihydroxy-isovalerate and 2-ketoisovalerate were generated in sequence by two *E. coli* endogenous genes *ilvC* and *ilvD*, encoding keto-acid reductoisomerase and dihydroxy-acid dehydratase respectively. Finally, isobutanol was generated from 2-ketoisovalerate by using two *Lactococcus lactis* enzymes, *kivd* encoding 2-keto acid decarboxylase and *adhA* encoding alcohol dehydrogenase. To facilitate the construction process, these five genes were overexpressed and distributed into plasmids pCL1920 and pTrc99a respectively. As AlsS is vital for the redirection of pyruvate into the isobutanol pathway, it was overexpressed in both plasmids. In addition, *acs* encoding the acetyl-CoA synthetase was also overexpressed in pCL1920 to improve the acetate assimilation. Thus, an entire pathway from acetate to isobutanol was constructed (Fig. 1).

In *E. coli*, *ydbK* encoding a putative pyruvate-ferredoxin oxidoreductase which can catalyze a reversible reaction between acetyl-CoA and pyruvate. Accordingly, we deduced acetate can be directly transformed into pyruvate via acetyl-CoA in wild *E. coli*. In other hand, the native pyruvate fermentation pathways, including pyruvate formate-lyase, pyruvate oxidase, aldehyde-alcohol dehydrogenase, and lactate dehydrogenase, which compete for pyruvate with isobutanol synthetic pathway were inactivated in turn in BW25113 to generate BWΔPPAL. Then, pCL1920-1 and pTrc99a-1 was co-transformed into BWΔPPAL to generate recombinant strain NH000. Unfortunately, no isobutanol accumulation was detected for NH000, and even no pyruvate was found in NH000 (data not shown). In previous report, isobutanol production could increase by up to 75% from acetate when pyruvate was added compared with the control [32]. Deficiency of intracellular pyruvate may result in no isobutanol synthesis of NH000. In addition, no lactate, formate and ethanol were detected in NH000 (data not shown), demonstrating the effective of blocking byproduct generation pathway.

### Isobutanol synthesis from acetate by overexpression of ***ydbK*** or anaplerotic pathway genes 

**Fig. 2:**
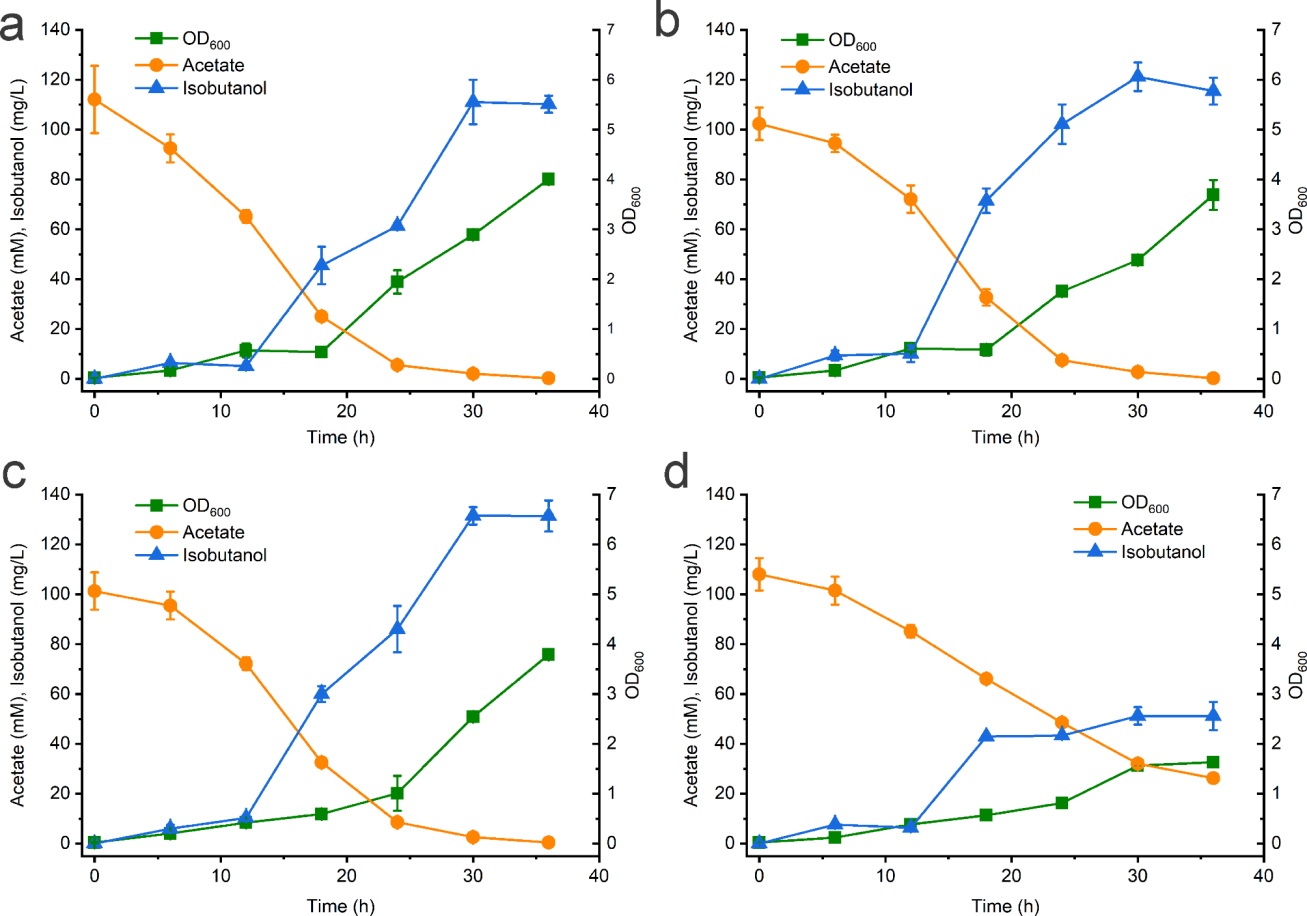
Batch fermentation of NH001 **(a)**, WY001 **(b)**, WY002 **(c)** and NH002 **(d)**. The error bars represent standard deviations from three replicate fermentations

To improve the pyruvate generation from acetate, the *ydbK* gene was inserted into plasmid pTrc99a-1 to generate pTrc99a-2 and co-transformed with pCL1920-1 into BWΔPPAL to generate NH001. As shown in Fig. 2a, recombinant strain NH001 showed a lag phase of 18 h. And then, the OD_600_ of NH001 began to increase quickly and reach a maximum OD_600_ 4.005 at 30 h. Acetate was completely consumed after 36 h batch fermentation. In addition, isobutanol accumulation was extremely low before 12 h and could reach a maximum isobutanol titer of 111.08 mg/L at 30 h. This phenomenon further suggested low intracellular expression level of *ydbK* and lack of pyruvate may result in no isobutanol accumulation in NH000. When the residual acetate was below 2 mM, isobutanol production of NH001 began to decrease. To our knowledge, this is the first report related to YdbK utilization in microbial production of chemicals from acetate.

Afterwards, we carefully analyzed the pyruvate metabolism and found pyruvate could also be generated from acetyl-CoA via two anaplerotic pathways in *E. coli* (Fig. 1). One is originated from decarboxylation of oxaloacetate by *pckA* encoding phosphoenolpyruvate carboxykinase. And then, phosphoenolpyruvate generated from oxaloacetate can convert into pyruvate through glycolytic pathway. The other pathway is responsible by NADP^+^-dependent malate dehydrogenase MaeB, which can transform malate into pyruvate [33]. It was reported these gluconeogenic pathways are highly activated when *E. coli* is grown with acetate, indicating that pyruvate could be supplemented by these two pathways [34]. Thus, *pckA* and *maeB* were inserted into pTrc99a-1 and then co-transformed with pCL1920-1 into BWΔPPAL to generate WY001. In batch cultivation, WY001 exhibited a maximum OD_600_ of 3.69, and the acetate assimilation curve between NH001 and WY001 were similar (Fig. 2b). In addition, isobutanol was increased quickly from 12 h and 121.21 mg/L of isobutanol could be obtained from 100 mM acetate sodium. As MaeB generate one NADPH by converting malate into pyruvate, overexpression of *maeB* was found be able to supply NADPH as well as pyruvate [35]. In addition, overexpression of *pckA* could increase intracellular level of phosphoenolpyruvate, a direct precursor of pyruvate. Accordingly, co-expression *maeB* and *pckA* exhibited positive effect on isobutanol production.

### Overexpression of transhydrogenase and NAD kinase further increased isobutanol titer of ***E. coli***

Although NADH-dependent alcohol dehydrogenase, such as AdhA from *L. lactis*, was recruited to reduce NADPH dependence in this study, one NADPH molecular is still required for one molecular isobutanol production. In wild *E. coli*, there are three pathways including pentose phosphate pathway, tricarboxylic acid cycle and membrane-bound transhydrogenase PntAB are responsible for generation of NADPH [33]. Among them, PntAB is a membrane-bound proton translocating pyridine nucleotide transhydrogenase which transfers a hydride from NADH to NADP^+^ with the concurrent production of NADPH and NAD^+^ [36, 37]. It was reported about 35–45% of NADPH applied in intracellular biosynthesis was supplied via PntAB [37]. Accordingly, improving the expression level of *pntAB* may be benefit for isobutanol production in *E. coli*, which has been proved in a previous report [38]. In addition, *yfjB* encoding NAD kinase in *E. coli*, can catalyze phosphorylation of NAD^+^ to NADP^+^ [39]. In previous report, it was found overexpression of *pntAB* and *yfjB* showed over 20% improvement in isobutanol titer for recombinant *E. coli* [28].

In this study, *pntAB* and *yfjB* were inserted into pCL1920-1 and then co-transformed with pTrc99a-3 into BWΔPPAL to generate WY002, and batch fermentation was performed for this strain. As shown in Fig. 2c, WY002 exhibited similar growth curve with WY001, indicating nearly no further metabolic burden occurred for overexpression of *yfjB* and *pntAB* compared with WY001. In addition, 131.5 mg/L isobutanol was detected at 30 h which was 8.49% higher than that of WY001. Song et al. also constructed a recombinant *E. coli* HM501::MAP for the production of isobutanol with acetate as carbon source. However, they found NADPH pool were not limiting factors for isobutanol production in *E. coli* when acetate was utilized [32]. Two reasons may be responsible for this discrepancy. Firstly, the engineering genes of HM501::MAP and WY002 in this work was quite different. In HM501::MAP, *fdh* from *C. boidinii* and *pntAB* from *E. coli* were employed for regeneration of NADPH, while *pntAB* and *yfjB* was overexpressed in WY002. Secondly, 5 mM of formate was supplemented into the medium when HM501::MAP was cultivated, while no further carbon source apart from acetate was used for WY002.

Considering no huge gap was exhibited for the isobutanol titers between NH001 and WY001, *pntAB* and *yfjB* were also over expressed in NH001 to generate NH002. And then batch fermentation were also performed for NH002. To our surprise, NH002 exhibited worse growth than NH001 indicated by the maximum OD_600_ of 1.635 at 36 h (Fig. 2d). In addition, only a 6 h rapid growth period was showed for NH002. The acetate assimilation rate was also obviously slower than NH001, WY001 and WY002. Especially, NH002 only produced 51.22 mg/L isobutanol at 30 h, representing only 42.25% of that of NH001. After 18 h, increase of isobutanol titer become very slow. These results indicated introduction of *pntAB* and *yfjB* into NH001 seriously affect its normal growth and isobutanol production, while overexpression of these two genes is advantage for WY001.

### Optimization of acetate concentration in the batch fermentation

**Fig. 3:**
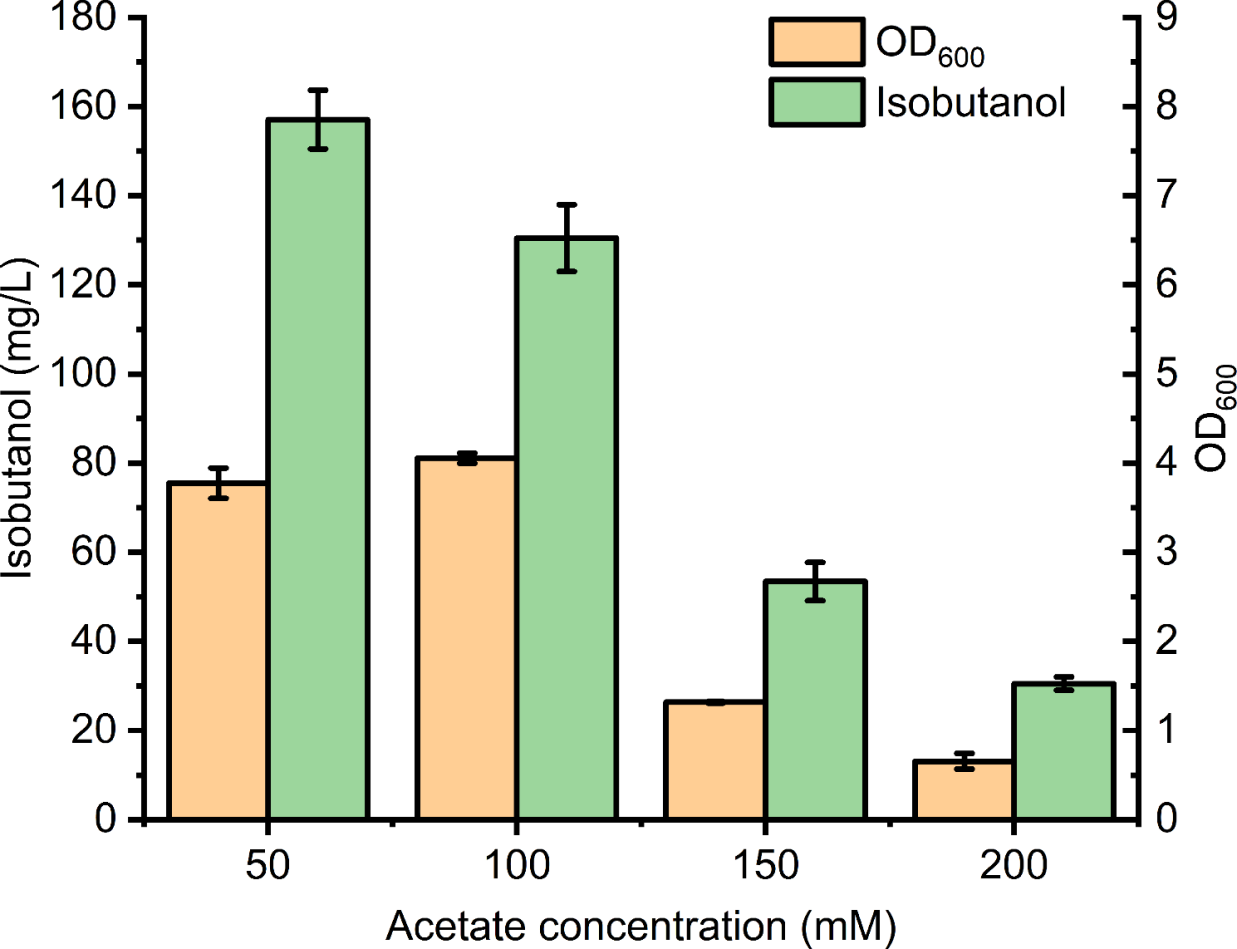
The effect of acetate concentration on strain growth and isobutanol production of WY002. The error bars represent standard deviations from three replicate fermentations

In a previous study, we found acetate is toxic to *E. coli* and inhibit its normal growth when the concentration of acetate exceeding 200 mM [17]. Considering different genes was engineered for WY002 compared with previous strains, the effect of acetate concentration on isobutanol production for WY002 was then explored. Four gradients of acetate, 50, 100, 150 and 200 mM were employed to perform batch cultivation of WY002. As shown in Fig. 3, Fig. S1 and Fig. S2, 150 and 200 mM of acetate interfered the growth seriously, indicated by the maximum OD_600_ of 0.83 and 0.38 respectively. As a result, the isobutanol production of WY002 was also very low for these two conditions. Although the final maximum OD_600_ were almost similar when 50 mM or 100 mM acetate was used for WY002, the growth curve were quite different (Fig. S1). When 50 mM acetate was used, WY002 could enter into logarithmic phase quickly and achieve the maximum OD_600_ of 3.774 at 24 h. In contrast, the OD_600_ of WY002 was only 1.01 at 24 and 42 h batch cultivation was needed to reach the maximum OD_600_ of 4.055. Consistent with growth curve, the maximum isobutanol production titer 157.05 mg/L of WY002 was also quickly achieved only for 18 h when 50 mM acetate was used as carbon source. To our knowledge, this was the highest isobutanol titer when acetate was selected as sole carbon source. Song et al. also constructed a recombinant *E. coli* to achieve isobutanol production from acetate, but the final strain HM501::MAP could only synthesize about 125 mg/L isobutanol after 120 h cultivation [32]. In addition, three compatible plasmids were employed to overexpress key genes which brought a heavy metabolic burden for host strain. Compared with HM501::MAP, WY002 engineered in this study could achieve 25.6% higher isobutanol production after only 18 h batch fermentation. Moreover, we also explored the effect of YdbK overexpression on isobutanol production in *E. coli* for the first time, which was not investigated by the report of Song et al. [32].

As 50 mM acetate exhibited higher isobutanol titer than that of 100 mM for WY002, NH001, WY001 and NH002 were also performed batch fermentation with 50 mM acetate as sole carbon source. As shown in Fig. S3, NH001, WY001 and NH002 showed a relatively quick growth when 50 mM acetate was employed and only 18–24 h was needed to achieve the maximum OD_600_. However, the maximum OD_600_ values of NH001, WY001 and NH002 were not obviously increased using 50 mM acetate. In addition, the isobutanol production titers of NH001, WY001 and NH002 were increased by 3.8%, 5.8% and 7.8% respectively when 50 mM acetate was used compared with these of the same strains using 100 mM acetate (Fig. S4). These results suggested 50 mM acetate could accelerate strain growth and slightly improve isobutanol production for NH001, WY001 and NH002 in batch fermentation.

## Conclusions

In this study, isobutanol production in *E. coli* with acetate as sole carbon source was investigated. Pyruvate could be generated from acetate via acetyl-CoA by pyruvate-ferredoxin oxidoreductase YdbK or anaplerotic pathways. To our knowledge, this is the first report about utilization of YdbK for isobutanol production. In addition, overexpression of transhydrogenase and NAD kinase coordinating with optimized acetate concentration further increased the isobutanol titer of WY002 to 157.05 mg/L, representing the highest isobutanol titer using acetate as sole carbon source.

However, compared with other carbon sources, such as glucose and glycerol, isobutanol titer from acetate as sole carbon source in *E. coli* is still slow and far from industrial production. Accordingly, further strain engineering should be carried out. For example, increasing the acetate tolerance will be benefit for strain biomass accumulation. As exhibited in this work, the maximum OD_600_ of recombinant strain were all below 4.5. Different strategies such as laboratory metabolic evolution [40], engineering of cobalamin-independent methionine synthase [41], rewiring global regulator cAMP receptor protein [42] and engineered global regulator H-NS [43] can be investigated to increase acetate tolerance in *E. coli*. In other hand, increasing the tolerance of isobutanol may also be advantageous for isobutanol production in *E. coli*. In a previous report, isobutanol-tolerant *E. coli* mutant obtained by evolution were capable of growth at 20 g/L isobutanol in glucose containing media [44]. Moreover, further improving the pyruvate transformation efficiency from acetate is crucial for isobutanol production. Regardless of YdbK or anaplerotic pathways, intracellular pyruvate titer may be not enough for strain growth and isobutanol production. As a result, exploration novel and effective pyruvate generation pathways from acetate as few enzymic reaction steps as possible is vital.

Considering high yields, high productivities are usually hard to achieve in cell-based systems due to the need to maintain life processes, a cell-free system containing enzymes in a bioreactor with continuous product removal is an alternative method for biofuel production. By using this technology, isobutanol from glucose at a maximum productivity of 4 g/L/h and a titer of 275 g/L with 95% yield could be achieved, which far exceed these parameters obtained by isobutanol producing strains [45]. However, NADPH, NADP, ATP and purified enzymes need to be added in the in-vitro production system, which will add the overall cost of isobutanol production. In comparison, direct fermentation of isobutanol from simple substrates by microbial cells may be a better choice for isobutanol production at present stage due to low cost, tolerance to complex environments and easy manipulation process.

## Materials and methods

### Bacterial strains

All strains, plasmids and oligonucleotides used in this study were listed in Table 1 and Table S1 respectively. *E. coli* BW25113 was employed for the construction of isobutanol producing strain. *E. coli* DH5α was selected as a base strain for the construction of recombinant plasmids.

**Table 1:** Strains and plasmids used in this study

Strains and Relevant characteristic plasmids		Source
**Strains**		
BW25113	*F*^*−*^, *λ*^*−*^, *rph-1*	Lab stock
DH5α	*F*^*−*^, *endA1*, *hsdR17* (*r*_*K*_^*−*^, *m*_*K*_^*+*^), *supE44*, *thi-l*, *l*^*−*^, *recA1*, *gyrA96*, Δ*lacU169 (Φ80lacZ* Δ*M15)*	Lab stock
BWΔPPAL	BW25113 (Δ*pflB*Δ*poxB*Δ*adhE*Δ*ldhA*)	This study
NH000	BWΔPPAL containing pTrc99a-1 and pCL1920-1	This study
NH001	BWΔPPAL containing pTrc99a-2 and pCL1920-1	This study
NH002	BWΔPPAL containing pTrc99a-2 and pCL1920-2	This study
WY001	BWΔPPAL containing pTrc99a-3 and pCL1920-1	This study
WY002	BWΔPPAL containing pTrc99a-3 and pCL1920-2	This study
**Plasmids**		
pKD3	*oriR6Kγ*, *bla*(Amp^R^), *cat*, *rgnB*(Ter)	[46]
pKD4	*oriR6Kγ*, *bla*(Amp^R^), *kan*, *rgnB*(Ter)	[46]
pTKRed	Spc^R^	[47]
pCP20	*bla* and *cat*, helper plasmid	[48]
pTrc99a	Amp^R^	Lab stock
pTrc99a-1	pTrc99a-*adhA*-*kivD*-*alsS*	This study
pTrc99a-2	pTrc99a-*adhA*-*kivD*-*alsS-ydbK*	This study
pTrc99a-3	pTrc99a-*adhA*-*kivD*-*alsS-pckA-maeB*	This study
pCL1920	Spc^R^	Lab stock
pCL1920-1	pCL1920-*alsS*-*ilvC*-*ilvD*-*acs*	This study
pCL1920-2	pCL1920-*alsS*-*ilvC*-*ilvD*-*acs-pntAB-yfjB*	This study

### Plasmids construction

The genes of *ilvC* and *ilvD*, encoding ketol-acid reductoisomerase and dihydroxy-acid dehydratase respectively, was fused together by overlap extension PCR. Firstly, the genome DNA of BW25113 and primers ilvC-QF/ilvC-QR were employed to obtain *ilvC* fragment. In the meantime, primers PCL-ilvD-NF and IlvD-ilvC-NR were used to obtain *ilvD* fragment. And then, *ilvC* and *ilvD* with homologous sequences were fused by two-step overlap extension PCR. Step 1: GoldenStar T6 DNA polymerase mix (TSINGKE Biological Technology) 45 µL, *ilvC* fragment 0.5 µL and *ilvC* fragment 0.5 µL. Cycling parameters: initial denaturation at 94 °C for 5 min, subsequent cycle steps containing 94 °C 30 s, annealing at 56 °C for 30 s, extension at 72 °C for 3.5 min, 10 cycles total, final extension at 72 °C for 10 min, and hold at 4 °C. Step 2: adding 2 µL of PCL-ilvD-NF and 2 µL of ilvC-QR into the tube. Cycling parameters: initial denaturation at 94 °C for 5 min, subsequent cycle steps containing 94 °C 30 s, annealing at 56 °C for 30 s, extension at 72 °C for 3.5 min, 25 cycles total, extension at 72 °C for 10 min, hold at 4 °C. All reactions were run with heated lid. The resulting 3.5 kb PCR product was analyzed electrophoresis in 0.8% agarose and performed DNA sequencing at TSINGKE Biological Technology. And then, *acs* gene encoding acetyl-CoA synthetase were fused with *ilvD-ilvC* fragment by overlap extension PCR. Primers Ilvc-acs-NF/Acs-PCL-NR were applied to amplify *acs* gene fragment containing homologous sequences with *ilvC*. Then, *ilvD-ilvC-acs* fragment with homologous sequences was assembled into pCL1920 by ClonExpress MultiS One Step Cloning Kit (Vazyme). Finally, *alaS* encoding acetolactate synthase from *B. subtilis* were directly synthesized by TSINGKE Biological Technology and ligated into the *Sal*I and *Bam*HI sites of pCL1920-*ilvD-ilvC-acs*. The resulting plasmid was named pCL1920-1.

Similarly, *alaS* encoding acetolactate synthase from *B. subtilis*, *kivd* encoding 2-keto acid decarboxylase from *L. lactis* and *adhA* encoding alcohol dehydrogenase from *L. lactis* was amplified and fused to an entire DNA fragment by overlap extension PCR. And then, *adhA-kivD-alsS* fragment was assembled with linear pTrc99a digested by *Sma*I site, resulting recombinant plasmid pTrc99a-1.

Next, *ydbK* encoding pyruvate-ferredoxin oxidoreductase was amplified by primers ydbk-zu-F/ydbk-zu-R and assembled with linear pTrc99a-1 digested by *Sma*I to generate pTrc99a-2. In the meantime, *pck* and *maeB* encoding phosphoenolpyruvate carboxykinase and malate dehydrogenase from *E. coli* respectively were firstly fused together, and then assembled with linear pTrc99a-1 digested by *Sma*I. As a result, recombinant pTrc99a-3 was generated.

**Fig. 4:**
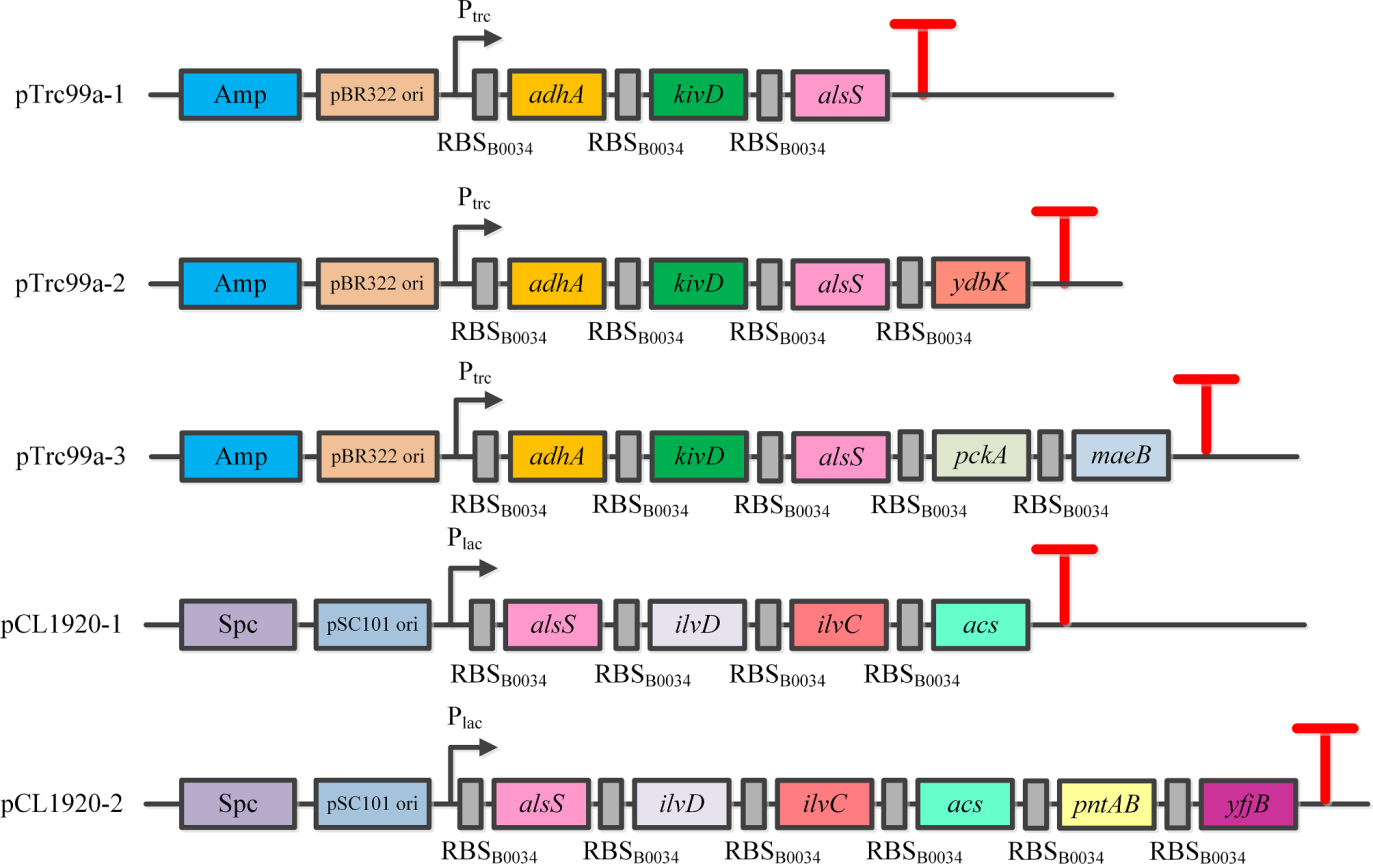
The organization of plasmids pTrc99a-1, pTrc99a-2, pTrc99a-3, pCL1920-1 and pCL1920-2

Then, pCL1920-1 was linear by PCR, using PCR-120-4-QF/PCR-120-4-QR as primers. And then, *pntA*, *pntB*, and *yfjB* encoding H+-translocating NAD(P) transhydrogenase subunit alpha, H+-translocating NAD(P) transhydrogenase subunit beta, and NAD kinase from *E. coli* respectively were fused by overlap extension PCR and assembled with linear pCL1920-1 to generate pCL1920-2. The organization of plasmids pTrc99a-1, pTrc99a-2, pTrc99a-3, pCL1920-1 and pCL1920-2 were shown in Fig. 4. The ribosome binding site RBS_B0034_ (AAAGAGGAGAAA) derived from Community RBS Collection (http://parts.igem.org/Ribosome_Binding_Sites/Prokaryotic/Constitutive/Community_Collection) was applied for genes overexpressed in plasmids.

### Gene deletion

The four genes, *pflB*, *poxB*, *adhE* and *ldhA* in *E. coli*, which encoding pyruvate formate-lyase, pyruvate oxidase, aldehyde-alcohol dehydrogenase, and lactate dehydrogenase respectively were knocked out in BW25113 sequentially by one-step inactivation method [46]. The resulting strain was named as BWΔPPAL. Finally, NH001 was generated by co-transforming pTrc99a-2 and pCL1920-1 into BWΔPPAL, NH002 was generated by co-transforming pTrc99a-2 and pCL1920-2 into BWΔPPAL, WY001 was generated by co-transforming pTrc99a-3 and pCL1920-1 into BWΔPPAL, WY002 was generated by co-transforming pTrc99a-3 and pCL1920-2 into BWΔPPAL and NH000 was generated by co-transforming pTrc99a-1 and pCL1920-1 into BWΔPPAL.

### Growth conditions

*E. coli* strains for cloning were cultivated in Luria-Bertani media (1% tryptone, 0.5% yeast extract and 1% NaCl) at 37 °C for 8–12 h. Different antibiotics were supplemented with appropriate concentrations, including ampicillin (100 mg/L), chloramphenicol (17 mg/L), kanamycin (25 mg/L), and spectinomycin (50 mg/L). For batch fermentation, a medium containing Na_2_HPO_4_ 33.9 g/L, KH_2_PO_4_ 15 g/L, NaCl 2.5 g/L, NH_4_Cl 5 g/L, MgSO_4_ 1mM, CaCl_2_ 0.1 mM, yeast extract 5 g/L was used, and sodium acetate was added with different concentration as indicated. 1 mL overnight cells were inoculated into 50 mL fermentation medium for batch fermentation, and strains were cultivated at 30 °C with 200 rpm shaking. Isopropyl β-D-1-thiogalactopyranoside (IPTG) was added at a final concentration of 0.2 mM, when the OD_600_ of *E. coli* cells reached 0.4–0.6.

### Analytical methods

High-performance liquid chromatography (Thermo Fisher Scientific, USA) equipped with a column of Aminex HPX-87 H ion exclusion particles (300 mm × 7.8 mm, Bio-Rad, Hercules, CA, USA) was used for determining sodium acetate concentration. The mobile phase was 5 mM sulfuric acid, with the flow rate of 0.6 mL/min and the column was maintained at 65 °C. Cell growth was monitored by OD_600_ using a UV5100H spectrophotometer (METASH, Shanghai China). The concentration of isobutanol was determined by gas chromatography (CROCKWAY, China) using an HP-FFAP column (25 m × 0.20 mm × 0.3 μm) (Agilent Technologies, USA) and a flame ionization detector (FID) described previously [32].

### Electronic supplementary material

Below is the link to the electronic supplementary material.

Supplementary Material 1

## Data Availability

All data generated and analyzed during this study were included in this manuscript.
