# Supplementary material for: Synthesis of isobutanol using acetate as sole carbon source in Escherichia coli
Source: Microb Cell Fact. 2023 Sep 27;22:196. doi: 10.1186/s12934-023-02197-w (PMC10537434; doi:10.1186/s12934-023-02197-w)
Supplement: Supplementary file 1 — Supplementary Material 1 [file 12934_2023_2197_MOESM1_ESM.docx]

Synthesis of isobutanol using acetate as sole carbon source in *Escherichia coli*

Pengfei Gu^1,&,*^, Shuo Zhao^1,&^, Hao Niu^1^, Chengwei Li^2^, Shuixing Jiang^2^, Hao Zhou^2^, Qiang Li^1^

^1^School of Biological Science and Technology, University of Jinan, Jinan 250022, People’s Republic of China

^2^RZBC GROUP CO., LTD., Rizhao 276800, Shandong, China

*Corresponding author: Pengfei Gu

Email: bio_gupf@ujn.edu.cn

Tel. : +86-531-82767364

Fax: +86-531-89736818

^&^ These authors have contributed equally to this work

**Table S1 Primers used in this study**

| Primers | Relevant characteristic |
| --- | --- |
| pflB-ZF | 5′-TGTCGAAGTACGCAGTAAATAAAAAATCCACTTAAGAAGGTAGGTGTTACGTGTAGGCTGGAGCTGCTTC-3′ |
| pflB-ZR | 5′-GTGGAGCCTTTATTGTACGCTTTTTACTGTACGATTTCAGTCAAATCTAAATGGGAATTAGCCATGGTCC-3′ |
| pflB-JF | 5′-TGAGCACAGTATCGCAAACAT-3′ |
| pflB-JR | 5′-ATTGCGGTGTTTCTCCAGATGTG-3′ |
| poxB-ZF | 5′-TCAGATGAACTAAACTTGTTACCGTTATCACATTCAGGAGATGGAGAACCGTGTAGGCTGGAGCTGCTTC-3′ |
| poxB-ZR | 5′-CATGGCATGTCCTTATTATGACGGGAAATGCCACCCTTTATGGGAATTAGCCATGGTCC-3′ |
| poxB-JF | 5′-ATACGGTGAGCAGCACAATGA-3′ |
| poxB-JR | 5′-GTCTATGGGTTGCGGTTGAATACTGCC-3′ |
| adhE-QF | 5′-ATTCGAGCAGATGATTTACTAAAAAAGTTTAACATTATCAGGAGAGCATTGTGTAGGCTGGAGCTGCTTC-3′ |
| adhE-QR | 5′-CCTTAACTGATCGGCATTGCCCAGAAGGGGCCGTTTATGTTGCCAGACAGCGCTACTGAATGGGAATTAGCCATGGTCC-3′ |
| adhE-JF | 5′-CACGTAATCAGTACCCAGAA-3′ |
| adhE-JR | 5′-AACGGTCGCATGAGCAGAAA-3′ |
| ldhA-ZF | 5′-GTAGCTTAAATGTGATTCAACATCACTGGAGAAAGTCTTGTGTAGGCTGGAGCTGCTTC-3′ |
| ldhA-ZR | 5′-ATTGGGGATTATCTGAATCAGCTCCCCTGGAATGCAGGGGAGCGGCAAGAATGGGAATTAGCCATGGTCC-3′ |
| ldhA-JF | 5′-ACAGCCCGAGCGTCATCAGCAGCGTCAA-3′ |
| ldhA-JR | 5′-ATGGTGTTATCGAGTTCATTAAGCTGCGGG-3′ |
| PCL-ilvD-NF | 5′-GCTTGCATGCCTGCAGGTCGACTCTAGAGGATCCCCAAGGAGATATACATATGCCTAAGTACCGTTCCGCCACCACCACTCATGGTCGT-3′ |
| Ilvd-ilvc-NR | 5′-GGCGCAGATTCAGTGTATTGAAGTAGTTAGCCATATGTATATCTCCTTTTAACCCCCCAGTTTCGATTTATCGCGCAC-3′ |
| Ilvc-acs-NF | 5′-GATATGAAACGTATTGCTGTTGCGGGTTAAAAGGAGATATACATATGAGCCAAATTCACAAACACACCATTCCTGCC-3′ |
| Acs-PCL-NR | 5′-TTGTAAAACGACGGCCAGTGAATTCGAGCTCGGTACCCTTACGATGGCATCGCGATAGCCTGCTTC-3′ |
| 1920-AlsS168-F | 5′-ACGCGTCGACAAGGAGATATACATATGTTGACAAAAGCAACAAAAGAAC-3′ |
| 1920-AlsS168-R | 5′-CGCGGATCCCTAGAGAGCTTTCGTTTTCATGAGTT-3′ |
| IlvC-QF | 5′-ATGGCTAACTACTTCAATACACTGAATCTGCG-3′ |
| IlvC-QR | 5′-TTAACCCGCAACAGCAATACGTTTCAT-3′ |
| PCR-1920-1-QF | 5′-CGAATTCACTGGCCGTCGTTTTACAACGTCGTGACTGGGAAAACCCTGGCG-3′ |
| PCR-1920-1-QR | 5′-AGCTCGGTACCCTTACGATGGCATCGCGATAGCCTGCTTCTCTTCAAG-3′ |
| 1920-pntA-QF | 5′-CTCAGTCCTAGGTATTATGCTAGCTACTAGAGAAAGAGGAGAAATACTAGATGCGAATTGGCATACCAAGAGAACGGTTAAC-3′ |
| 1920-pntA-QF1 | 5′-AAGAGAAGCAGGCTATCGCGATGCCATCGTAAGGGTACCGAGCTTTTACAGCTAGCTCAGTCCTAGGTATTATGCTAG-3′ |
| PntA-pntB-QR | 5′-CAATGTATGCAGCTGTAACTAATCCTCCAGACATATGTATATCTCCTTTTAATTTTTGCGGAACATTTTCAGCATG-3′ |
| PntB-QF | 5′-ATGTCTGGAGGATTAGTTACAGCTGCATACATTGTTGCCGC-3′ |
| PntB-QR | 5′-TTACAGAGCTTTCAGGATTGCATCCACGCTGGCT-3′ |
| PntB-yfjB-QF | 5′-AGCCAGCGTGGATGCAATCCTGAAAGCTCTGTAAAAGGAGATATACATATGAATAATCATTTCAAGTGTATTGGCATTGTGGGA-3′ |
| yfjB-1920-QR | 5′-TTTTCCCAGTCACGACGTTGTAAAACGACGGCCAGTGAATTCGTTAGAATAATTTTTTTGACCAGCCGAGCTTGGTG-3′ |
| Pnt-3-JF | 5′-GATTGCCGAAGCCGCCGTAGTAG-3′ |
| Pnt-3-JR | 5′-CATCCGCATTAAAATCTAGCGAGGGC-3′ |
| P-adhA-NF | 5′-CAGACCATGGAATTCGAGCTCGGTACCCAAGGAGATATACATATGAAAGCAGCAGTAGTAAGACACAA-3′ |
| AdhA-kivD-NR | 5′-CGGTCTAATAGGTAATCTCCTACTGTATACATATGTATATCTCCTTTTATTTAGTAAAATCAATGACCATTCGGCC-3′ |
| adhA-QF | 5′-ATGAAAGCAGCAGTAGTAAGACACAATCCA-3′ |
| adhA-QR | 5′-TTATTTAGTAAAATCAATGACCATTCGGCCTTC-3′ |
| Kivd-alsS-NF | 5′-GCTGAACAAAATAAATCATAAAAGGAGATATACATATGTTGACAAAAGCAACAAAAGAACAAAAATCCC-3′ |
| alsS-P-NR | 5′-TGCAGGTCGACTCTAGAGGATCCCCATGTATATCTCCTTCTAGAGAGCTTTCGTTTTCATGAGTTCCCCG-3′ |
| ydbK-zu-F | 5′-AAACGAAAGCTCTCTAGGGGGATCCTCTAGAGAAGGAGATATACATATGATTACTATTGACGGTAATGGCGCGGTTGC-3′ |
| ydbK -zu-R | 5′-AAAACAGCCAAGCTTGCATGCCTGCAGGTCGATTAATCGGTGTTGCTTTTTTCCGCTTTTCC-3′ |
| 99A1-pckA-F | 5′-AAACGAAAGCTCTCTAGGGGGATCCTCTAGAGAAGGAGATATACATATGCGCGTTAACAATGGTTTGACCCCGCAAGA-3′ |
| pckA-maeB-R | 5′-TCAAGTGCACTTTGTTTTAACTGGTCATCCATATGTATATCTCCTTTTACAGTTTCGGACCAGCCGCTACCAGC-3′ |
| maeB-zu-F | 5′-ATGGATGACCAGTTAAAACAAAGTGC-3′ |
| maeB-99A1-R | 5′-AAAACAGCCAAGCTTGCATGCCTGCAGGTCGATTACAGCGGTTGGGTTTGCGCTTCTACCACGG-3′ |
| 99A1-ZU-JF | 5′-ACGGCGGTTTCTTATTCTCA-3′ |
| 99A1-ZU-JR | 5′-CGGCGGATTTGTCCTACTCA-3′ |
| PCR-1920-1-QF | 5′-CGAATTCACTGGCCGTCGTTTTACAACGTCGTGACTGGGAAAACCCTGGCG-3′ |
| PCR-1920-1-QR | 5′-AGCTCGGTACCCTTACGATGGCATCGCGATAGCCTGCTTCTCTTCAAG-3′ |





**Fig. S1** The growth curve of WY002 in batch fermentation under different concentrations of acetate. The error bars represent standard deviations from three replicate fermentations





**Fig. S2** The isobutanol production of WY002 in batch fermentation under different concentrations of acetate. The error bars represent standard deviations from three replicate fermentations





**Fig. S3** The growth curve of NH001, WY001 and NH002 in batch fermentation under 50 mM of acetate. The error bars represent standard deviations from three replicate fermentations





**Fig. S4** The isobutanol production of NH001, WY001 and NH002 in batch fermentation under 50 mM of acetate. The error bars represent standard deviations from three replicate fermentations
